# Association between Poor Sleep Quality and Subsequent Peptic Ulcer Recurrence in Older Patients with Mild Cognitive Impairment: Examining the Role of Social Engagement

**DOI:** 10.1038/s41598-019-38715-3

**Published:** 2019-02-18

**Authors:** Boye Fang, Shuyan Yang, Ruirui Xu, Gengzhen Chen

**Affiliations:** 1 University of Hong Kong, Pokfulam, Hong Kong; 2 Hong Kong Baptist University, Kowloon Tong, Hong Kong; 3 Shantou University Medical College, Shantou, China

## Abstract

This study aims to examine whether perceived poor sleep quality predicts subsequent recurrence of peptic ulcer disease (PUD) in older patients with mild cognitive impairment following *Helicobacter pylori* eradication and to investigate whether social engagement status alters this association. Of 1,689 older patients with *H*. *pylori*-infected PUD recruited from eight Grade-A hospitals in the People’s Republic of China between 2011 and 2014, *H*. *pylori* was eradicated and PUD cleared in 1,538 patients by the end of 2014; 1,420 of these were followed for up to 36 months. The Kaplan–Meier method was used to compare the proportion of PUD recurrence, as confirmed with esophagogastroduodenoscopy, among older patients with different levels of sleep quality and social engagement statuses. Multivariate Cox-proportional hazards models were performed to examine the association between sleep quality and PUD recurrence, and the role of social engagement in altering this relationship. The results showed that PUD recurrence was more prevalent in poor (10.8%) compared with good sleepers (5.5%). However, increased and continued social engagement reduced the proportion to 7.2% and 8.2% among poor sleepers, respectively. Poor sleep quality was associated with subsequent PUD recurrence (hazard ratio [HR] 1.965 (1.002, 3.518)). However, no significant difference was observed between good and poor sleepers who reported increased (HR 1.428 (0.736, 2.380)) and continued (HR 1.273 (0.915, 2.492)) social engagement, suggesting that increased and continued social engagement prevented the effect of poor sleep quality on PUD recurrence. To conclude, poor sleep quality is associated with subsequent PUD recurrence. However, increased and continued social engagement may moderate this association.

## Introduction

Poor sleep quality^1^ and peptic ulcer disease (PUD)^2^ are public health issues that reduce quality of life among older adults. Partially attributed to age-related increases in comorbid conditions, medication use, and sleep structure changes, poor sleep quality is found in over one third of older adults worldwide^3^. PUD, which is defined as peptic injury of the digestive tract leading to a mucosal break reaching the submucosa that usually occurs in the stomach and/or proximal duodenum^4^, is particularly prevalent among older adults^2^. Although treatment to eradicate *Helicobacter pylori* has effectively reduced the rate of PUD recurrence from over 40% to 5–25%^5–7^, a 2-year prospective study concluded that only a minority of *H*. *pylori*-reinfected patients experienced PUD recurrence and that none of the patients who experienced PUD recurrence screened positive for *H*. *pylori* reinfection^7^. This suggests the existence of alternative pathways in the course of PUD recurrence.

Indeed, emerging clinical evidence has suggested that sleep quality may have an impact on gastrointestinal health. During deeper sleep stages, defensive factors against PUD development and recurrence such as gastric bicarbonate efflux, gastric mucosal blood flow, and melatonin secretion were found to increase, while aggressive mediators such as gastrin secretion decreased^8–10^. These protective systems can be impaired by poor sleep quality, including difficulties falling asleep, multiple nighttime awakening, and early waking^10,11^. In particular, decreased prostaglandin levels and blood supply to the gastric mucosa resulting from aging can further increase the risk of PUD and complications in elderly poor sleepers^2,12^. Increased psychological stress was also observed in older adults who experienced sleep disturbances^12^, which further affects gastroduodenal acid secretion and impairs the biological defence against ulcerogenic agents^11–13^. Consistently, available empirical findings have suggested that older patients (≥80 years) with PUD experienced poorer sleep quality than their counterparts without PUD^13^. A more recent study further showed that women who slept for less than 7 h/day were nearly twice as likely to develop PUD than those who slept for more than 9 h/day^12^. However, the cross-sectional design of these studies cannot test the temporal association between sleep quality and PUD. Additionally, these studies solely depended on retrospective self-reports for information regarding PUD occurrence and did not specify whether PUD referred to the first ulcer onset or recurrent ulcer(s), which further undermine the validity of the reported associations.

The Activity Theory argues that social engagement is important for achieving positive adjustment in old age, and that continued engagement in meaningful social activities helps maintain positive attitude and good health condition^14^. Consistently, a number of studies have suggested a positive relationship between social engagement and improved physical and psychological wellbeing in older people^14–16^. Therefore, it is likely that maintenance of social engagement plays a role in the association between poor sleep quality and subsequent PUD recurrence, which has yet been tested in exiting literature. To address these limitations, this study analysed 3-year longitudinal data to examine the long-term effect of poor sleep quality on subsequent PUD occurrence in older adults and evaluated the role of social engagement in altering this trajectory.

## Methods

### Baseline

dong Province of the People’s Republic of China. From January 2011 to October 2014, older patients (≥55 years) with mild cognitive impairmentdong Province of the People’s Republic of China. From January 2011 to October 2014, older patients (≥55 years) with mild cognitive impairment (MCI) diagnosed with *H*. *pylori*-infected PUD were referred to the baseline study. MCI was determined based on available clinical and neuropsychological information and was made by consensus of a panel of neuropsychologists, neurologists, and psychiatrists according to the international guidelines of Petersen criteria^17^. A peptic ulcer refers to any circumscribed break of ≥5 mm in diameter with apparent depth covered with exudates occurring in the duodenum or stomach as determined by endoscopic examination conducted using the same type of endoscope (GIF-XQ260, Olympus Optical Co., Ltd., Tokyo, Japan). *H*. *pylori* infection was confirmed by positive rapid urease test (RUT; Ballard Medical Products, Draper, UT, USA) and histological examination. Severity of inflammation was graded using the Sydney System^18^.

Patients with previous *H*. *pylori* eradication treatments, malignancy, previous gastroduodenal operation, Zollinger–Ellison syndrome, or who had received anti-ulcer treatment or ulcerogenic drugs (i.e., nonsteroidal anti-inflammatory drugs, or anticoagulant and antiplatelet drugs) in the past 3 months were excluded.

Among the 2,080 patients that met the inclusion criteria, 1,689 (81.2%) who provided informed consent were successfully recruited. These patients received a 10-day anti-*pylori* treatment with esomeprazole (20 mg b.i.d.), amoxicillin (1000 mg b.i.d.), and clarithromycin (500 mg b.i.d.), followed by a 4-week anti-ulcer therapy with omeprazole (20 mg b.i.d.). A confirmation test was conducted 4 weeks after discontinuation of the anti-ulcer therapy to avoid false-negative results. Negative RUT results were considered indicative of successful eradication of *H*. *pylori*. Healing of ulcers was confirmed by endoscopic and histological examinations.

### Follow-up

By the end of 2014, 1,538 (91.1%) patients achieved *H*. *pylori* eradication and healed PUD. Four weeks later, these patients were invited to have an esophagogastroduodenoscopy (EGD) examination to detect PUD recurrence and a RUT and pathological examination to determine *H*. *pylori* reinfection at 6-month intervals for up to 36 months, regardless of the presence or absence of PUD symptoms. Of these patients, 118 failed to complete the follow-up study due to the use of ulcerogenic (n = 48) or anti-ulcer (n = 20) medications, receipt of gastroduodenal operation (n = 12) or sleep treatments (n = 24), presence of malignancy (n = 5), failure of contact (n = 3), missing data (n = 4), and death (n = 2). No significant differences were found between participants and non-participants regarding gender and age. A minimal sample size of 1,054 was required to achieve a statistical power of 0.9 to detect an effect size of 0.2 at a probability level of 0.05. This study was approved by the ethics committee of Shantou University Medical College. All procedures performed in studies involving human participants were in accordance with the ethical standards of the institutional and/or national research committee and with the 1964 Helsinki declaration and its later amendments or comparable ethical standards.

### Measurements

Outcome variable. Recurrent PUD was defined as the presence of recurrent active- or healing-stage ulcers in the duodenum or stomach at least 6 months after the initial diagnosis as confirmed by EGD.

Major independent variables. Self-perceived sleep quality was assessed using the 19-item self-administered Pittsburgh sleep quality index (PSQI), covering seven components of sleep quality (subjective sleep quality, sleep duration, sleep latency, habitual sleep efficiency, sleeping medication use, sleep disturbances, and daytime dysfunction) in the preceding month^19^. With a potential range of 0–21, a score of <5 and ≥5 indicated good and poor sleep quality, respectively. The PSQI has been demonstrated to exhibit adequate psychometric properties in its previous use in older Chinese individuals^19^. In this study, the PSQI displayed an internal reliability alpha of 0.78. The PSQI was measured at 6-month intervals and an average score was used for subsequent analysis.

Patients screened with poor sleep quality were further examined for changes in engagement in the following social activities: (1) interacting with relatives, friends, or neighbours, (2) participating in hobby groups (such as playing chess, Mahjong, cards, or other hobby groups), (3) attending sports groups (such as dancing, fitness, Qigong, Tai Chi, yoga, or other sports), (4) attending community-related organisations, and (5) doing voluntary or charity work. These items have been used in previous research of older Chinese individuals, with good psychometric properties reported^20^. Patients were then categorised into five mutually exclusive groups: Group 1 (with good sleep quality), Group 2 (with poor sleep quality and increased social engagement: at least one more activity at endpoint than at baseline), Group 3 (with poor quality and continued social engagement: the same number of activities at baseline and at endpoint that were greater than 0), Group 4 (with poor sleep quality and decreased social engagement: at least one fewer activity at endpoint than at baseline), and Group 5 (with poor sleep quality but without social engagement: no engagement in any activity at either baseline or endpoint).

Control variables. Sociodemographic characteristics were retrieved from the patients’ medical records, including age, gender, body mass index, socioeconomic status (whether the patient is living under local poverty line), previous ulcer locations (gastric, duodenal, and gastric-duodenal), and chronic conditions (cardiovascular disease, cerebrovascular disease, diabetes mellitus, renal disease, and liver disease). Excessive alcohol consumption was defined as >175 g/week for men and >105 g/week for women. Cigarette smoking was defined as ≥20 cigarettes/week. Self-rated depressive symptoms were assessed using the 10-item Chinese version of Center for Epidemiological Studies Depression Short Form (CES-D)^21^. Participants rated the frequency of which they had experienced each of the listed mood and behavioural symptoms in the past week (0 = rarely or none of the time, 1 = some of the time, 2 = most of the time). Two items indicating a positive affect were reversely coded. With a potential range of 0–18, a higher sum score indicates a higher severity of depressive symptoms. The Chinese CED-D has displayed desirable validity and reliability in previous research^21^. This study recorded an internal alpha of 0.82 for the CED-D. The CED-D was measured at 6-month intervals and an average score was used for subsequent analysis. The seven-item Generalized Anxiety Disorder Assessment (GAD-7) was used to assess the severity of generalised anxiety disorder (GAD)^22^. The participants rated the frequency of which they had experienced each of the listed symptoms of GAD in the past two weeks (0 = not at all, 1 = several days, 2 = more than half the days, 3 = nearly every day). With a potential summed score ranging from 0 to 21, a higher score suggests a greater severity of GAD and the threshold score of 10 indicates GAD. The Chinese GAD-7 is available and has demonstrated adequate psychometric properties in previous research^22^. Cognitive impairment was assessed using the Chinese version of the Mini-Mental State Examination (CMMSE), which has shown adequate psychometric properties in the diagnosis of dementia^23^. With a potential range of 0–30, a higher score indicates better cognitive function. The CMMSE has an internal alpha of 0.839 in this study.

### Data analysis

Data were analysed using SPSS 21.0 for Windows (SPSS Inc., Chicago, IL, USA). Sample characteristics and endoscopic findings were summarised as descriptive data. Continuous variables were described as means ± standard deviation (SD) and tested with analyses of variance after normality check. Categorical variables were presented as ns (%) and assessed with the chi-square test. Multivariate Cox-proportional hazards models were performed to investigate the association between poor sleep quality and subsequent PUD recurrence, controlling for sociodemographic variables and significant confounders in bivariate analyses (Model 1). In Model 2, an interaction term of poor sleep quality × change in social engagement status was developed to ascertain whether changes of engagement in social activities may moderate the relationship between poor sleep quality and subsequent PUD recurrence. To further test this potentially moderating effect, an additional model was constructed to evaluate the difference of PUD recurrence risk among Groups 1, 2, 3, 4, and 5, as described in the previous section. Cumulative probabilities of PUD recurrence among groups were analysed using the Kaplan–Meier method. Prior to the performance of Multivariate Cox-proportional Hazards Models and Kaplan–Meier Curves, proportionality test was conducted and the predictors were found to satisfy proportional hazard assumption, suggesting that proportional hazard assumption was not violated. A p-value of <0.05 was considered statistically significant.

## Results

### Sample characteristics and PUD recurrence

Over half of the patients at baseline were male (51.6%), with a mean age of 68.70 years (SD = 8.66) (Table 1). The results of the chi-square test revealed that, at baseline, living under the local poverty line, cardiovascular disease, excessive alcohol consumption, depressive symptoms, GAD, and poor sleep quality were more common in the PUD recurrence (+) group than in the PUD recurrence (−) group (Table 1). At follow-up, the proportion of *H*. *pylori* reinfection was significantly higher (67.8%) in the PUD recurrence (+) group than in the PUD recurrence (−) group (7.8%).

**Table 1:** Sample Characteristics and Endoscopic Findings in Older Patients (n = 1,420).

Baseline Characteristics	Total	PUD Recurrence (+)	PUD Recurrence (−)	P-value
Number	1,420	118	1,302	
Sociodemographic factors				
Age	68.70 ± 8.66	71.62 ± 9.86	68.71 ± 8.50	0.062
Female gender	687 (48.4%)	57 (48.3%)	630 (48.4%)	0.078
Body mass index (BMI)	23.6 ± 3.2	23.7 ± 3.3	23.6 ± 3.2	0.727
Living under local poverty line	213 (15.0%)	23 (19.5%)	190 (14.6%)	0.042
Pre-existing peptic ulcer locations				0.568
Gastric	550 (38.7%)	45 (38.1%)	505 (38.8%)	
Duodenal	834 (58.7%)	69 (58.5%)	765 (58.8%)	
Gastric-duodenal	36 (2.5%)	4 (3.4%)	32 (2.5%)	
Medical conditions				
Cardiovascular disease	393 (27.7%)	43 (36.4%)	350 (26.9%)	0.009
Cerebrovascular disease	341 (24.0%)	32 (27.1%)	309 (23.7%)	0.058
Diabetes mellitus	426 (30.0%)	39 (33.1%)	387 (29.7%)	0.055
Renal disease	111 (7.8%)	10 (8.5%)	101 (7.8%)	0.091
Liver disease	253 (17.8%)	20 (16.9%)	233 (17.9%)	0.098
Cognitive status (C-MMSE)	21.85 ± 4.76	21.07 ± 5.18	22.09 ± 4.08	0.627
Cigarette smoking	398 (28.0%)	33 (28.0%)	365 (28.0%)	0.259
Excessive alcohol consumption	94 (6.6%)	22 (18.6%)	72 (5.5%)	<0.001
Psychological factors				
Depressive symptoms	326 (23.0%)	47 (39.8%)	279 (21.4%)	<0.001
Generalized Anxiety Disorders	369 (26.0%)	37 (31.4%)	332 (25.5%)	0.027
Sleep quality (0–21)	7.03 ± 4.41	11.73 ± 4.43	6.58 ± 4.12	<0.001
Good (0–4)	852 (60.0%)	56 (47.5%)	796 (61.1%)	
Poor (5–21)	568 (40.0%)	62 (52.5%)	506 (38.9%)	
*Poor sleep quality* + *increased social engagement*	218 (15.4%)	17 (14.4%)	201 (15.4%)	
*Poor sleep quality* + *continued social engagement*	182 (12.8%)	16 (13.6%)	166 (12.7%)	
*Poor sleep quality* + *decreased social engagement*	108 (7.6%)	18 (15.3%)	90 (6.9%)	
*Poor sleep quality* + *no social engagement*	60 (4.2%)	11 (9.3%)	49 (3.8%)	
Follow-up characteristics				
*H*. *pylori* reinfection	182 (12.8%)	80 (67.8%)	102 (7.8%)	<0.001
Duration of follow-up	34.62 ± 3.17	33.88 ± 3.68	34.83 ± 3.01	0.126

^†^Data presented as number (%), mean (SD).

### Proportion of PUD recurrence

**Figure 1:**
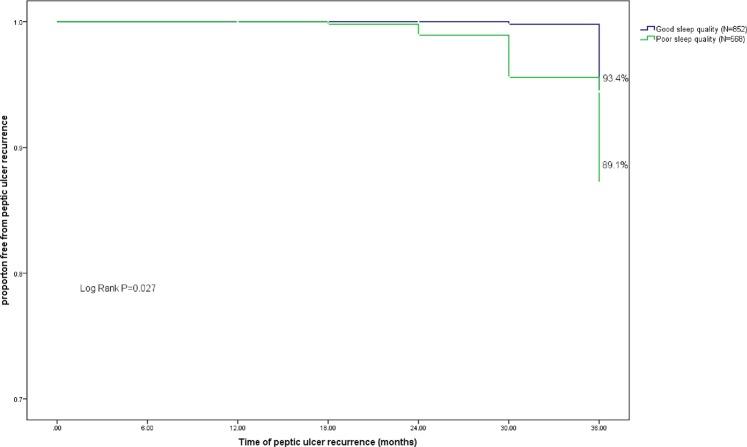
Kaplan-Meier survival analysis comparing peptic ulcer recurrence in patients with different levels of sleep quality. Cumulative incidence curves show the differences of time and peptic ulcer recurrence events in patients with (1) good sleep quality and (2) poor sleep quality. P-value presents the statistical difference between the two groups in log-rank test.

**Figure 2:**
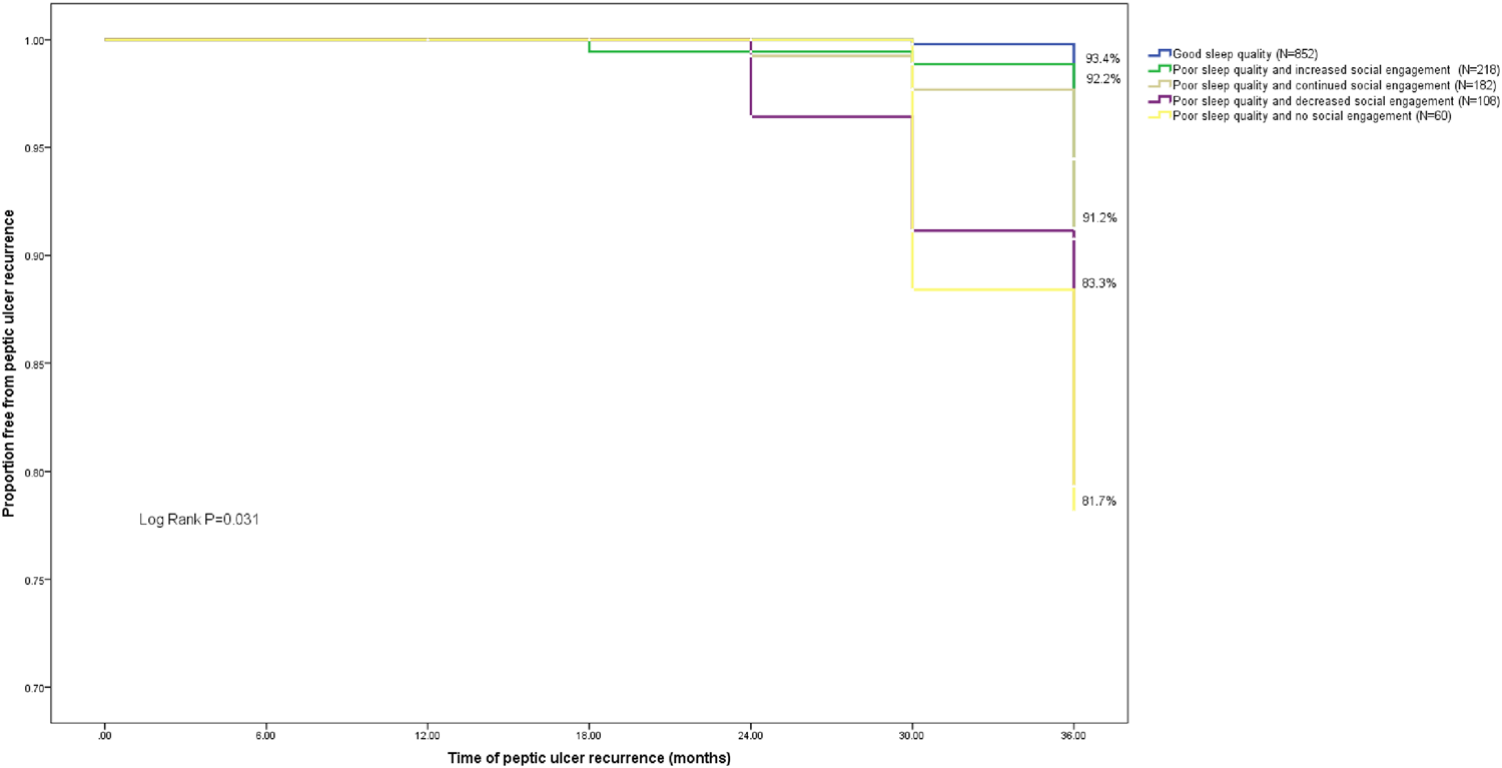
Kaplan-Meier survival analysis comparing peptic ulcer recurrence in patients with different levels of sleep quality and change of social engagement. Cumulative incidence curves show the differences of time and peptic ulcer recurrence events in patients with (1) good sleep quality, (2) poor sleep quality and increased social engagement, (3) poor sleep quality and continued social engagement, (4) poor sleep quality and decreased social engagement, and (5) poor sleep quality and no social engagement. P-value presents the statistical difference between the five groups in log-rank test.

The EGD results revealed an overall 36-month cumulative PUD recurrence proportion of 8.2% (n = 118, annual recurrence incidence = 2.7%) in this study. Sixty patients with PUD recurrence reported symptoms such as stomachache (n = stomach ache (n = 20), acid reflux (n = 18), nausea (n = 9), feeling of fullness (n = 8), and heartburn (n = 5), while others remained asymptomatic. The proportion of PUD recurrence was higher in patients with poor sleep quality (10.8%) than in those with good sleep quality (5.5%) (Fig. 1). Among patients with poor sleep quality, increased and continued social engagement reduced the proportion of PUD recurrence to 7.2% and 8.2%, respectively. By contrast, a higher proportion of PUD recurrence was observed in poor sleepers who reported decreased (16.3%) or lack of social engagement (17.8%) (Fig. 2).

### Association between poor sleep quality and subsequent PUD recurrence

Multivariate Cox-proportional hazards model (Model 1 in Table 2) showed that poor sleep quality increased the risk of PUD recurrence (hazard ratio [HR] 1.965 (1.002, 3.518)), after adjusting for a set of covariates. Other factors associated with subsequent PUD recurrence were socioeconomic status (living under the local poverty line), the presence of cardiovascular disease, depressive symptoms, and GAD at baseline, as well as *H*. *pylori* reinfection at follow-up.

**Table 2:** Interaction Effect between Poor Sleep Quality and Change in the Level of Social Engagement on Subsequent Peptic Ulcer Recurrence (n = 1,420).

Variables	Multivariate Model 1^a^		Multivariate Model 2^b^	
	HR (95%CI)	P-value	HR (95%CI)	P-value
Demographic characteristics				
Age	1.739 (0.969, 3.295)	0.263	1.635 (0.872, 2.591)	0.190
Female gender	0.901 (0.728, 1.239)	0.180	0.891 (0.690, 1.282)	0.219
Body mass index (BMI)	1.392 (0.931, 2.308)	0.120	1.293 (0.849, 2.971)	0.271
Socioeconomic status (Living under local poverty line)	1.298 (1.013, 2.308)	0.018	1.271 (1.001, 2.510)	0.028
Bacteriologic factors				
*H*. *pylori* reinfection	2.621 (1.410, 5.226)	<0.001	2.695 (1.672, 4.059)	˂0.001
Behavioral factors				
Excessive alcohol consumption	1.523 (0.941, 2.082)	0.082	1.387 (0.950, 2.167)	0.092
Medical condition				
Cardiovascular disease	1.859 (1.256, 2.825)	<0.001	1.756 (1.136, 2.767)	0.003
Psychological factors				
Depressive symptoms	2.079 (1.195, 3.608)	0.028	2.062 (1.175, 3.552)	0.046
Generalized Anxiety Disorders	1.816 (0.916, 3.228)	0.085	1.756 (0.862, 3.174)	0.086
Change in the levels of social engagement				
Decreased	2.023 (1.218, 3.208)	0.019	1.958 (1.022, 3.027)	0.020
Unchanged	1.668 (1.021, 2.075)	0.045	1.518 (0.977, 2.013)	0.069
Increased	1.268 (0.766, 3.159)	0.227	1.194 (0.657, 2.938)	0.271
None (ref.)				
Sleep Quality				
Poor	1.965 (1.002, 3.518)	0.026	1.906 (1.008, 3.139)	0.046
Good (ref.)				
Interactions				
Poor sleep quality × decreased level of social engagement			2.008 (1.169, 3.366)	0.016
Poor sleep quality × unchanged level of social engagement			1.836 (1.050, 3.079)	0.038
Poor sleep quality × decreased level of social engagement			1.566 (1.008, 2.989)	0.049
Poor sleep × no social engagement (ref.)				

^a^Multivariate Model 1 includes age, female gender, BMI, socioeconomic status, H. pylori reinfection, cardiovascular disease, excessive alcohol consumption, depressive symptoms, Generalized Anxiety Disorders, and change in the levels of social engagement as covariates, and poor sleep quality as the major independent variable.

^b^Multivariate Model 2 includes age, female gender, BMI, socioeconomic status, H. pylori reinfection, cardiovascular disease, excessive alcohol consumption, depressive symptoms, Generalized Anxiety Disorders as covariates; poor sleep quality and change in the levels of social engagement as the main effect; and poor sleep quality × change in the levels of social engagement as the tested interaction effect.

### Moderating effect of change in social engagement

Among the 1,420 patients, 852 (60.0%) reported good sleep quality (Group 1). Of the 568 (40.0%) patients who reported poor sleep quality, 218 (15.4%) reported increased social engagement (Group 2), 182 (12.8%) reported continued social engagement (Group 3), 108 (7.6%) reported decreased social engagement (Group 4), and 60 (4.2%) reported no social engagement (Group 5). To test whether specific changes of engagement in social activities may moderate the association between poor sleep quality and subsequent PUD recurrence, an interaction term of poor sleep quality × change in social engagement status was added into Model 2 (Table 2). The results showed that poor sleep quality × decreased level of social engagement (HR 2.008 (1.169, 3.366)), poor sleep quality × unchanged level of social engagement (HR 1.836 (1.050, 3.079)), and poor sleep quality × decreased level of social engagement (HR 1.566 (1.008, 2.989)) were significant interaction terms. To further understand the potentially moderating effect of the change in social engagement, additional multivariate analyses were conducted to assess the subsequent risk of PUD recurrence in these five groups, with Group 1 as the reference group (Model 3, Table 3). The results indicated no significant differences between Group 1 and either Group 2 (HR 1.428 (0.736, 2.380)) or Group 3 (HR 1.273 (0.915, 2.492)), suggesting that although poor sleep quality augmented the subsequent risk of PUD, poor sleepers who increased or maintained social engagement did not necessarily follow this trend. Increased or maintained social engagement helped prevent the effect of poor sleep quality on PUD recurrence. Compared with Group 1, Group 4 (HR 1.539 (1.008, 2.568)) and Group 5 (HR 1.887 (1.013, 3.429)) were subject to a higher risk of PUD recurrence, suggesting that poor sleepers with reduced or lack of social engagement were more likely to experience PUD recurrence than good sleepers.

**Table 3:** Moderating Effect of the Change in the Levels of Social Engagement on the Association between Poor Sleep Quality and Subsequent PUD Recurrence (n = 1,420).

	Multivariate Model 3^a^	
Variables	HR (95%CI)	P-value
Sociodemographic factors		
Age	1.629 (0.860, 2.783)	0.179
Female gender	0.902 (0.672, 1.361)	0.271
Body mass index (BMI)	1.312 (0.865, 2.734)	0.086
Socioeconomic status (living under local poverty line)	1.297 (1.008, 2.684)	0.027
Bacteriologic factors		
*H*. *pylori* reinfection	2.767 (1.582, 4.588)	0.006
Behavioral factor		
Excessive alcohol consumption	1.388 (0.980, 1.132)	0.076
Medical condition		
Cardiovascular disease	1.830 (1.225, 2.728)	0.004
Psychological factors		
Depressive symptoms	2.058 (1.171, 3.548)	0.043
Generalized Anxiety Disorders	1.752 (0.858, 3.170)	0.089
Groups based on sleep quality and change of social engagement		
Group 1^b^ (ref.)		
Group 2^c^	1.428 (0.736, 2.380)	0.382
Group 3^d^	1.273 (0.915, 2.492)	0.083
Group 4^e^	1.539 (1.008, 2.568)	0.025
Group 5^f^	1.887 (1.013, 3.429)	0.043

^a^Multivariate Model 3 includes age, female gender, BMI, socioeconomic status, *H*. *pylori* reinfection, cardiovascular disease, excessive alcohol consumption, depressive symptoms, Generalized Anxiety Disorders as covariates, and groups based on sleep quality and change in the levels of social engagement as the major independent variable.

^b^Group 1: Participants with good sleep quality.

^c^Group 2: Participants with poor sleep quality and increased social engagement.

^d^Group 3: Participants with poor sleep quality and continued social engagement.

^e^Group 4: Participants with poor sleep quality and decreased social engagement.

^f^Group 5: Participants with poor sleep quality and no social engagement.

## Discussion

This study yielded an overall 36-month cumulative proportion of PUD recurrence of 8.2% and an annual incidence of 2.7%. This is higher than the annual incidence of 1.9% documented in Japanese patients with a mean age of 56.5 years and followed for 4 years^6^, but lower than the annual incidence of 5.5% reported in a 24-month observation based on a clinical sample (mean age = 55.1 years) in Taiwan^7^. Discrepancies in the rate of PUD recurrence may be attributed to methodological variations, such as the length of observation period and patient selection.

The present data tentatively suggest a higher risk of PUD recurrence in older patients with poor sleep quality, especially when the poor sleepers are also subject to reduced or lack of social engagement. The pathway underlying the association between poor sleep quality and PUD recurrence remains inconclusive. One plausible explanation is that gastric mucosal blood flow, which may accelerate ulcer healing, can increase during sleep^11,12^. Gastric acid secretion has also been found to decrease during rapid eye movement sleep, possibly resulting from a decrease in plasma noradrenaline and histamine release during this stage of sleep^9,13^. However, compromised sleep quality is likely to impair these protective mechanisms against repeated gastric mucosal injuries^11^.

Another probable explanation may be associated with the autonomic and enteric nervous systems. The gut-brain axis functions through mechanisms involving intestinal permeability, enteroendocrine signalling, and immune activation, which is important for regulating the digestive tract and maintaining the gut immune system^24^. However, sleep disturbances and circadian rhythm disorders might interfere with its normal function, potentially altering gastrointestinal susceptibility to ulcerogenic agents^11^. Experimental evidence has also suggested that poor sleep quality is likely to generate depression^24,25^, which has also been found as a significant factor associated with PUD recurrence in this study. Depression can stimulate psychological stress, which may dysregulate sympathoadrenal stress response mechanisms and the hypothalamic-pituitary-adrenal axis^24^. It is possible that such neuroendocrinological abnormalities can further impair gastroduodenal function and alter acid secretions^10^, which might contribute to an elevated risk of PUD recurrence.

Melatonin, a hormone produced by the pineal gland that regulates sleep and wakefulness, might also affect the association between poor sleep quality and PUD recurrence. Melatonin has been found to serve as a potent stimulant of bicarbonate that can accelerate ulcer healing by inhibiting gastric acid secretion, increasing gastric mucosal blood flow, and interfering with prostaglandin-dependent pathways^25^. Additional evidence has suggested that melatonin can protect against PUD recurrence by scavenging free radicals and accelerating mucosal microcirculation and cell proliferation^10^. However, poor sleep quality can lead to melatonin deficiency, which might further interfere with the potential protective effect of melatonin on PUD recurrence^10^.

Finally, sleep disturbance and deficiency can activate the release of proinflammatory cytokines such as interleukin (IL)-1, IL-6, and IL-8, which might in turn activate inflammatory cells in patients with *H*. *pylori*-induced ulcers^26^. Meanwhile, recent evidence has suggested that elevated expression levels of endothelial growth factor C in gastric mucosal inflammation induced by sleep apnea, sleep loss, and nighttime hypoxia are likely to increase the risk of developing PUD^27,28^.

A previous study reported no association between regular participation in sport groups and self-reported PUD development and recurrence^29^. Possibly due to methodological differences in terms of studied population, use of measurements, and assessment, this study yielded inconsistent results. Specifically, this study noted that increased and continued social engagement altered the impact of poor sleep quality on subsequent PUD recurrence, suggesting that heterogeneous patterns may exist in the association between sleep quality and gastroduodenal diseases depending on social engagement status. It is plausible that regular social engagement may improve sleep quality, as suggested by recent empirical findings^30^, which in turn might diminish the risk of PUD. Alternatively, older adults with poor sleep quality who maintain socially active tend to have better accessibility to social support, which may allow them to better cope with the psychological stress and depressive affect associated with poor sleep^31^, thus potentially decreasing the risk of stress-related gastroduodenal symptoms^32^, such as PUD and its recurrence.

The association between poor sleep quality and an elevated risk of subsequent PUD recurrence suggests the importance of providing proper sleep treatments to previous ulcer patients. In many countries around the world, even in places where non-pharmaceutical therapies are recommended as initial treatment (i.e. U.S and Canada), pharmacotherapy remains the mainstream therapeutic approach for sleep-related problems^33^. This is particularly true in China, where there is a significant shortage of qualified therapists for providing non-pharmacological sleep treatments^34^. Although non-pharmacological treatment such as cognitive behavioural therapy (CBT) has been shown to be effective and is recommended as an initial treatment for chronic primary and comorbid insomnia by the American Academy of Sleep Medicine^34^, pharmacotherapy remains the mainstay treatment for sleep disturbances in China^33^. Hence, it will be important to develop a systematic treatment protocol for older adults affected by sleep problems, especially those with previous PUD. Such a systematic protocol should consider integrating pharmacotherapy with CBT, which has demonstrated effectiveness in increasing remission and treatment response and reducing PSQI scores among older adults^35^. Some evidence has shown that eszopiclone, zolpidem, and ramelteon improved sleep outcomes in older adults; however, pharmacotherapy alone cannot be an ultimate and long-term solution^35^. Meanwhile, issues such as previous treatment responses, patient preference, comorbid conditions, contraindications, concurrent drug-drug interactions, and potential side effects should be considered when using pharmacotherapy^34^. Alternatively, given a higher proportion of PUD recurrence in patients with poor sleep quality, it is necessary to also regularly screen for PUD recurrence in poor sleepers who previously experienced PUD. Doing so requires interdisciplinary cooperation between geriatric psychiatrists and gastroenterologists.

Additionally, our findings that social engagement altered the impact of poor sleep quality on subsequent PUD recurrence empirically support the Activity Theory, which posits that older adults who maintain or initiate meaningful social activities are more likely to remain physically healthy^14^. Therefore, it is necessary for geriatric psychiatrists to routinely ask older patients with poor sleep quality about their social engagement levels and identify patients with social isolation or diminishing supportive networks. It is equally important for them to become familiar with available intervention resources in the local community, and make referrals when necessary. For older adults with more serious cognitive impairment, their primary caregivers should also be interviewed to obtain more reliable information. Considering the shortage of formal later-life social activities in China^31^, a promising approach to improve the accessibility and quality of social engagement among older Chinese individuals is to develop multidisciplinary teams including physicians, psychiatrists, social workers, policy makers, and other community participants working together in a coordinated manner.

This study has several limitations. First, lacking experimental design means causal relationships cannot be confirmed between the predictors and PUD recurrence. Specifically, although this study suggests that poor sleep quality is associated with a higher risk of PUD recurrence in older patients with MCI, it is alternatively likely that psychological stress related to the experience of PUD symptoms might have contributed to a greater likelihood of poor sleep quality, which denotes the possibility of bi-directionality of the present results. Future studies with more rigorous experimental design are needed to verify the direction of this relationship. Second, although we have collected a relatively large sample for analysis, the dependence on convenience sampling means that the present results cannot be generalised to other older populations with MCI. Future empirical analyses using representative data are needed to re-examine the present results. Third, although the measurement of sleep quality was administered by experienced and well-trained geriatric psychiatrists, the nature of the self-reporting approach is inherently subject to bias. Future research using data based on objective measurements of sleep quality may improve our understanding of the specific association revealed in this study. Lastly, although we have included a wide range of potential associated factors for PUD recurrence in the multivariate models, certain potential predictors such as stress, life adversity, and personality trait were not tested, as they were not available in our database. These limitations should be addressed in future empirical investigation.

## Conclusions

This study tentatively revealed the differential longitudinal relationships between self-perceived poor sleep quality and subsequent PUD recurrence among older individuals with MCI, depending on social engagement status. Older adults with poor sleep quality appear to experience a higher risk of PUD than those with good sleep quality. However, increased or continued social engagement may alter this association by protecting older poor sleepers against PUD recurrence. These findings imply that the risk of PUD recurrence among older people with MCI may be reduced by improving sleep quality and providing easier access to meaningful social activities. If these findings are replicated in future studies using epidemiological data and experimental design, it will lead to an enhanced understanding of the roles of sleep quality and social engagement in PUD recurrence, which may shed light on the mechanism of this disease and help to identify new pathways for the prevention and treatments of PUD recurrence.

## References

[1] Bao YP (2017). Cooccurrence and bidirectional prediction of sleep disturbances and depression in older adults: Meta-analysis and systematic review. Neurosci. Biobehav. Rev..

[2] Seo JH (2016). Long-term recurrence rates of peptic ulcers without helicobacter pylori. Gut Liver..

[3] Suzuki K, Miyamoto M, Hirata K (2017). Sleep disorders in the elderly: Diagnosis and management. Gen Fam Med..

[4] Lanas A, Chan FKL (2017). Peptic ulcer diseases. Lancet..

[5] Lau, J.Y. *et al*. Systematic Review of the Epidemiology of Complicated Peptic Ulcer Disease: Incidence, Recurrence, Risk Factors and Mortality. *Digestion*. **84**, 102–113 (2011). 10.1159/000323958 21494041

[6] Miwa H (2004). Recurrent peptic ulcers in patients following successful helicobacter pylori eradication: a multicenter study of 4940 patients. Helicobacter..

[7] Tseng GY (2007). Recurrence of peptic ulcer in uraemic and non-uraemic patients after helicobacter pylori eradication: a 2-year study. Aliment Pharmacol Ther..

[8] Cai H (2015). Sleep duration and mortality: a prospective study of 113138 middle-aged and elderly Chinese men and women. Sleep..

[9] Knutsson AN, Bøggild H (2010). Gastrointestinal disorders among shift workers. Scand J Work Environ Health..

[10] Kato K (2002). Circadian rhythm of melatonin and prostaglandin in modulation of stress-induced gastric mucosal lesions in rats. Aliment Pharmacol Ther..

[11] Khanijow V (2015). Sleep Dysfunction and Gastrointestinal Diseases. Gastroenterol Hepatol..

[12] Ko SH, Baeg MK, Ko SY, Han KD (2016). Women Who Sleep More Have Reduced Risk of Peptic Ulcer Disease; Korean National Health and Nutrition Examination Survey (2008–2009). Sci. Rep..

[13] Räihä M, Seppälä O, Impivaara MT, Hyyppä LR, Knuts LS (1994). Chronic illness and subjective quality of sleep in the elderly. Aging Clin Exp Res..

[14] Park NS (2009). The Relationship of Social Engagement to Psychological Well-Being of Older Adults in Assisted Living Facilities. J Appl Gerontol..

[15] Thoits PA (2011). Mechanisms Linking Social Ties and Support to Physical and Mental Health. J Health Soc Behav..

[16] Jeste DV (2013). Association Between Older Age and More Successful Aging: Critical Role of Resilience and Depression. Am J Psychiatry..

[17] Petersen RC (1999). Mild cognitive impairment: clinical characterization and outcome. Arch Neurol..

[18] Dixon MF, Genta RM, Yardley JH, Correa P (1996). Classification and grading of gastritis. The updated Sydney System. International workshop on histopathology of gastritis, Huston 1994. Am J Surg Pathol..

[19] Yue J, Huang C, Wu H, Dong BR (2013). Association of sleep quality and dementia among long-lived Chinese older adults. AGE..

[20] Fu C, Li Z, Mao Z (2018). Association between Social Activities and Cognitive Function among the Elderly in China: A Cross-Sectional Study. Int. J. Environ. Res. Public Health..

[21] Silverstein M, Cong Z, Li S (2006). Intergenerational Transfers and Living Arrangements of Older People in Rural China: Consequences for Psychological Well-Being. J Gerontol B Psychol Sci Soc Sci..

[22] Liu S (2016). Caregiver burden and prevalence of depression, anxiety, and sleep disturbances in Alzheimer’s disease caregivers in China. J Clin Nurs..

[23] Zhang ZX, Hong X, Li H (1999). The Mini-Mental State Examination in population aged 55 years and over in urban and rural areas of Beijing. Chinese Journal of Nervous and Mental Disorders..

[24] Young EA, Abelson JL, Cameron OG (2004). Effect of comorbid anxiety disorders on the hypothalamic–pituitary–adrenal axis response to a social stressor in major depression. Biol Psychiatry..

[25] Celinski K (2011). Effects of melatonin and tryptophan on healing of gastric and duodenal ulcers with Helicobacter pylori infection in humans. J Physiol Pharmacol..

[26] Haghazali M (2011). Proinflammatory cytokines and thrombomodulin in patients with peptic ulcer disease and gastric cancer, infected with Helicobacter pylori. Indian J Pathol Microbiol..

[27] Schulz R, Hummel C, Heinemann S, Seeger W, Grimminger F (2002). Serum Levels of Vascular Endothelial Growth Factor Are Elevated in Patients with Obstructive Sleep Apnea and Severe Nighttime Hypoxia. Am J Respir Crit Care Med..

[28] Taghizadeh S (2014). Expression levels of vascular endothelial growth factors a and c in patients with peptic ulcers and gastric cancer. J Gastric Cancer..

[29] Cheng Y, Macera CA, Davis DR, Blair SN (2000). Physical activity and peptic ulcers. West J Med..

[30] Friedman EM, Hayney MS, Love GD (2005). Social relationships, sleep quality, and interleukin-6 in aging women. PNAS..

[31] Lou V, Chi I, Kwan C, Leung A (2013). Trajectories of social engagement and depressive symptoms among long-term care facility residents in Hong Kong. Age Ageing..

[32] Levenstein S, Rosenstock S, Jacobsen RK, Jorgensen T (2015). Psychological stress increases risk for peptic ulcer, regardless of helicobacter pylori infection or use of nonsteroidal anti-inflammatory drugs. Clin Gastroenterol Hepatol..

[33] Morin CM, Colecchi C, Stone J, Sood R, Brink D (1999). Behavioral and Pharmacological Therapies for Late-Life Insomnia A Randomized Controlled Trial. JAMA..

[34] Chung KF, Lee CKY (2002). Over-the-counter sleeping pills: a survey of use in Hong Kong and a review of their constituents. Gen Hosp Psychiatry..

[35] Schutte-Rodi S, Broch L, Buysse D, Dorsey C, Sateia M (2008). Clinical Guideline for the Evaluation and Management of Chronic Insomnia in Adults. J Clin Sleep Med..

